# Pediatric antimicrobial stewardship in the COVID-19 outbreak

**DOI:** 10.1017/ice.2020.312

**Published:** 2020-06-24

**Authors:** Eneritz Velasco-Arnaiz, Maria Goretti López-Ramos, Silvia Simó-Nebot, Iolanda Jordan, María Ríos-Barnés, Mireia Urrea-Ayala, Manuel Monsonís, Clàudia Fortuny, Antoni Noguera-Julian

**Affiliations:** 1 Sant Joan de Déu Antimicrobial Stewardship Program (SJD-ASP), Sant Joan de Déu Hospital, Barcelona, Spain; 2 Infectious Diseases Unit, Department of Pediatrics, Sant Joan de Déu Hospital, Barcelona, Spain; 3 Pharmacy Department, Sant Joan de Déu Hospital, Barcelona, Spain; 4 Centre for Biomedical Network Research on Epidemiology and Public Health (CIBERESP), Madrid, Spain; 5 Pediatric Intensive Care Unit, Sant Joan de Déu Hospital, Barcelona, Spain; 6 Department of Pediatrics, University of Barcelona, Barcelona, Spain; 7 Red de Investigación Translacional en Infectología Pediátrica, RITIP, Madrid, Spain; 8 Infection Control Department, Sant Joan de Déu Hospital, Barcelona, Spain; 9 Clinical Microbiology Department, Sant Joan de Déu Hospital, Barcelona, Spain

*To the Editor—*Growing evidence supports the positive impact of antimicrobial stewardship programs (ASPs) on antimicrobial use, including pediatrics.^1^ Although short of the level of acceptance these have reached in the United States, the implementation of pediatric ASPs in European hospitals has increased over the last few years.^1^

It has been suggested that the ASP should be helpful in the preparation for and response to the SARS-CoV-2/COVID-19 outbreak,^2^ but no formal recommendations have been published. Whether pediatric ASP remains an essential activity or not during the COVID-19 pandemic has yet to be clarified. Here, we describe how the COVID-19 pandemic has impacted antimicrobial use in a referral pediatric hospital, and we propose a supporting role for ASP teams in the local management of the outbreak.

The first COVID-19 case in Catalonia, Spain, was reported on February 25, 2020. By mid-March, most pediatric and obstetrics departments in the region were shut to increase the capacity for adult COVID-19 patients. Hospital Sant Joan de Déu Barcelona (SJD) remained the largest pediatric and maternal referral center in the region. COVID-19 and non–COVID-19 pediatric and young adult patients were transferred to our wards and pediatric ICU (PICU), and the number of daily deliveries tripled, whereas all nonemergency clinical, teaching, and research activities were postponed. Compared to the same months in 2019, in March 2020, total hospital stays decreased by 0.8% in the PICU and 15.2% in non-PICU areas, and in April 2020, total hospital stays decreased by 23.7% in the PICU and 22.2% in non-PICU areas.

Following institutional recommendations, the SJD-ASP^3^ team reduced on-site work, but they continued to provide specific recommendations on individual antimicrobial prescriptions upon consultation by prescribers, and they monitored systemic antibiotic and antifungal use: days of-therapy (DOT) per 100 days present (DP). From March 16 to April 30 2020, 210 randomly selected prescriptions were assessed for quality.^3^

Because SARS-CoV-2 is a viral infection, it is not expected to directly influence antibiotic or antifungal use beyond the use of antibiotics with possible antiviral effect (ie, azithromycin)^4^ and the use of broad-spectrum antibiotics for superinfection in severe COVID-19 patients.^5^ However, we also observed antimicrobial use changes indirectly related to the outbreak. Antimicrobial use in March and April 2020 was significantly higher than in the same months in 2019 (Table 1). As expected, the use of azithromycin, included as first-line therapy in severe COVID-19 patients in combination with hydroxychloroquine, increased, particularly in the PICU. The use of ceftriaxone and teicoplanin, which were also prescribed at admission in severe COVID-19 cases, doubled in the PICU in April 2020 compared with April 2019. Other than ceftriaxone, antibiotics for community-acquired infections were prescribed less than in the same period in 2019, and cefazolin use decreased due to the dramatic drop in the number of surgeries. In contrast, the use of most broad-spectrum anti–gram-negative drugs with anti-*Pseudomonas* activity rose in the PICU, and piperacillin-tazobactam and ciprofloxacin use also increased in non-PICU patients. These changes were probably related to the transfer of patients with immunosuppressive and/or other complex conditions from other centers that had shut their pediatric departments. Similarly, the rise in micafungin use and, to a lesser extent, voriconazole use, was likely related to the transfer of oncological and hematopoietic stem cell transplantation (HSCT) patients already on antifungal prophylaxis or antifungal treatment in accordance with other institutions’ local protocols. The temporary modification of our antifungal prophylaxis protocol for oncology patients (inhaled liposomal amphotericin plus oral fluconazole) meant minimizing the use of nebulized drugs and led to an increase in intravenous liposomal amphotericin use.

**Table 1.** Total Antimicrobial Use (AU) and Selected Antibiotic (Ab) and Antifungal (Af) Use in February, March, and April 2019 and 2020, in Days of Therapy (DOT) per 100 Days Present (DP) in PICU and Non-PICU Areas (Excluding Operating Rooms)^a^

		February (DOT/100 DP)			March (DOT/100 DP)			April (DOT/100 DP)		
	Year	Non-PICU	PICU	Total	Non-PICU	PICU	Total	Non-PICU	PICU	Total
AU (Ab+Af)	2019	62.9	109.2	172.1	63.3	91.9	155.2	63.3	82.8	146.0
	2020	−0.4	−10.0	−10.4	+6.6	+6.6	+13.2	−1.4	+56.9	+55.5
Total Antibiotics	2019	57.4	103.5	160.9	57.4	89.6	147.0	57.2	79.5	136.7
	2020	−0.8	−9.9	−10.7	+4.5	−2.9	+1.6	−2.0	+37.5	+35.5
Total Antifungals	2019	5.5	5.7	11.2	5.9	2.3	8.2	6.1	3.2	9.3
	2020	+0.5	−0.2	+0.3	+2.1	+9.5	+11.6	+0.6	+19.4	+19.9
**Selected Antibiotics**										
Amikacin	2019	1.1	6.1	7.2	0.4	1.5	1.9	0.7	0.5	1.2
	2020	−0.3	−4.6	−4.9	+0.8	−0.7	+0.1	+0.2	+0.8	+1.0
Amoxicillin	2019	3.8	2.6	6.4	3.4	4.6	8.0	4.3	0.5	4.8
	2020	+0.7	−1.0	−0.3	+0.6	−1.1	−0.5	−2.1	+5.3	+3.2
Amoxicillin/clavulanate	2019	11.2	12.9	24.0	12.7	9.6	22.3	12.7	8.5	21.2
	2020	−0.9	+0.4	−0.6	−2.0	−2.1	−4.1	−5.4	−2.3	−7.7
Ampicillin	2019	5.2	1.3	6.5	4.2	5.1	9.3	3.6	1.4	5.0
	2020	−1.9	+0.8	−1.1	−0.7	−2.6	−3.3	−1.4	+0.2	−1.2
Azithromycin	2019	1.5	3.3	4.8	2.1	4.8	6.9	2.2	6.0	8.1
	2020	+0.2	+1.2	+1.4	+0.9	+4.2	+5.1	+0.4	+4.1	+4.5
Cefazolin	2019	1.4	17.9	19.2	1.9	15.8	17.8	2.1	15.7	17.8
	2020	+1.4	−4.4	−3.0	+0.3	−2.5	−2.2	−1.1	−2.5	−3.6
Cefotaxime	2019	2.3	12.4	14.7	2.8	6.8	9.6	1.3	9.7	11.0
	2020	−0.8	−4.1	−4.9	−1.0	−2.4	−3.4	+0.6	−3.5	−2.9
Ceftazidime	2019	0.7	5.0	5.7	0.6	0.8	1.4	0.2	0.9	1.1
	2020	+0.1	−4.7	−4.7	+0.2	0	+0.2	+0.3	−0.9	−0.6
Ceftriaxone	2019	5.2	1.7	7.0	5.4	2.6	8.1	5.8	1.4	7.1
	2020	−1.3	+1.7	+0.4	0	+0.2	+0.2	+0.1	+1.8	+1.8
Ciprofloxacin	2019	1.2	3.1	4.2	1.3	4.5	5.7	1.5	6.8	8.3
	2020	+0.2	+1.7	+1.9	+0.6	−2.0	−1.4	+1.3	+0.3	+1.7
Linezolid	2019	0.1	3.1	3.1	0.2	2.3	2.5	0.3	1.0	1.3
	2020	+0.3	+0.7	+1.0	−0.1	−2.1	−2.3	−0.2	−0.1	−0.4
Meropenem	2019	5.0	3.7	8.7	4.0	2.8	6.8	5.1	4.6	9.7
	2020	−2.2	−1.2	−3.4	−0.9	+0.9	−0.1	−1.9	+4.8	+2.9
Piperacillin/tazobactam	2019	2.3	6.8	9.0	1.2	10.7	12.0	1.9	4.9	6.9
	2020	+1.4	+0.7	+2.0	+3.2	+2.9	+6.2	+2.6	+12.3	+14.9
Teicoplanin	2019	0.1	0.4	0.5	0.2	1.0	1.2	0.2	1.2	1.4
	2020	+0.7	+0.6	+1.3	+0.7	+2.0	2.7	+0.2	+7.1	+7.3
Vancomycin	2019	3.8	15.0	18.8	3.1	10.9	14.0	3.3	7.2	10.5
	2020	+0.3	−2.0	−1.7	+2.4	−1.4	+1.0	+0.7	+0.9	+1.5
**Selected Antifungals**										
Fluconazole	2019	1.6	0.7	2.2	0	2.3	2.3	2.6	3.2	5.8
	2020	+1.9	−0.4	+1.5	+2.7	−1.6	+1.1	−2.4	−3.2	−5.7
Liposomal amphotericin	2019	1.6	0.4	2.0	1.7	0	1.7	1.5	0	1.5
	2020	−0.7	+1.0	+0.4	+1.5	+1.0	+2.5	+1.8	+1.6	+3.4
Micafungin	2019	0.6	3.9	4.6	0	0	0	0	0	0
	2020	+0.1	−0.7	−0.6	+0.1	+7.0	+7.1	+0.3	+13.2	+13.5
Posaconazole	2019	1.0	0	1.0	0.3	0	0.3	1.2	0	1.2
	2020	−0.5	+0.4	0	+1.2	+0.3	+1.5	+1.0	+0.7	+1.6
Voriconazole	2019	0.7	0.7	1.4	1.4	0	1.4	0.4	0	0.4
	2020	−0.3	−0.7	−1.0	−1.0	+2.8	+1.8	+0.2	+7.2	+7.3

Note. Ab, antibiotic; Af, antifungal; AU, antimicrobial use; DOT, days of therapy; DP, days present; PICU, pediatric intensive care unit.

a Year 2020 data are given as increases or decreases compared to the same month in year 2019.

Despite major changes in antimicrobial use, we have not observed a critical deterioration of antimicrobial prescription quality to date. Of the 210 evaluated prescriptions, 167 (79.5%) were considered ‘optimal’ in accordance with current protocols, compared with 316 of 400 (79.0%) in the same period in 2019. However, continuous monitoring allowed the identification of high workload areas deserving enhanced support, like involving the care of oncology patients.

In the context of a pandemic, changing clinical circumstances may negatively affect the quality of antimicrobial prescriptions; prescribers have to work outside their comfort zone, dealing with a new disease^6^ and/or ‘unusual’ patient profiles (ie, young adults or pregnant women in a PICU in our case). New COVID-19 protocols are constantly updated in accordance with newly available information, and previous protocols have been temporarily modified in favor of medical nonsurgical management of some conditions (eg, noncomplicated appendicitis).^7^

Our data show that the SARS-CoV-2 pandemic has the potential to have a significant impact on antimicrobial use in the pediatric inpatient population; pediatric ASP monitoring and interventions remain useful to preserve the quality of prescriptions, at least in the short term. However, the COVID-19 pandemic is still ongoing, as are other non–COVID-19 health issues such as AMR,^5^ so healthcare resource distribution and organization in the post–COVID-19 period are uncertain. Planning the response to epidemic waves should include the widespread integration of ASP, with (1) involvement of the ASP team in guidelines development as exemplified in “Multicenter Initial Guidance on Use of Antivirals for Children with COVID-19/SARS-CoV-2”^8^; (2) integrated response of common ASP local and external partnerships and infrastructures, including structured interviews, formularies, and other information technology tools^9^; and (3) coordination of indicator selection and monitoring routines to support a continuous evaluation strategy.

If pediatric ASPs have suffered some weakening in the current crisis, they should be reinforced promptly to sustain high-quality care, maintaining the principles of antibiotic stewardship.^5^ The potential benefit of a more active role for pediatric ASPs in the outbreak response, above and beyond their regular activities, should be taken into consideration.
